# Prognostic factors and validation of the histologic chronicity score for C3 glomerulopathy: a registry analysis

**DOI:** 10.1093/ckj/sfae077

**Published:** 2024-03-20

**Authors:** Safak Mirioglu, Egemen Cebeci, Halil Yazici, Ulver Derici, Gulizar Sahin, Ganime Coban, Necmi Eren, Ozkan Gungor, Fatih Dede, Tamer Dincer, Kultigin Turkmen, Taner Basturk, Murat Duranay, Hakki Arikan, Onur Tunca, Omer Celal Elcioglu, Erhan Tatar, Zeki Aydin, Deren Oygar, Serap Demir, Mehmet Tanrisev, Ilhan Kurultak, Aysegul Oruc, Aydin Turkmen, Omer Faruk Akcay, Hakki Cetinkaya, Savas Ozturk, the Glomerular Diseases Working Group of the Turkish Society of Nephrology (TSN-GOLD), Yasemin Ozluk, Ipek Isik Gonul, Gulistan Gumrukcu, Cigdem Vural, Emine Kilinc Gunay, Aysel Colak, Iclal Gurses, Haci Hasan Esen, Ayse Aysim Ozagari, Saba Kiremitci, Handan Kaya, Cigdem Ozdemir, Funda Tasli, Arzu Saglam Ayhan, Yasemin Yuyucu Karabulut, Neslihan Guney, Ufuk Usta, Berna Aytac Vuruskan

**Affiliations:** Division of Nephrology, Bezmialem Vakif University Faculty of Medicine, Istanbul, Turkey; Division of Nephrology, Istanbul Haseki Training and Research Hospital, University of Health Sciences, Istanbul, Turkey; Division of Nephrology, Istanbul University Istanbul Faculty of Medicine, Istanbul, Turkey; Division of Nephrology, Gazi University Faculty of Medicine, Ankara, Turkey; Division of Nephrology, Sultan 2. Abdulhamid Han Training and Research Hospital, University of Health Sciences, Istanbul, Turkey; Department of Pathology, Bezmialem Vakif University Faculty of Medicine, Istanbul, Turkey; Division of Nephrology, Kocaeli University Faculty of Medicine, Kocaeli, Turkey; Division of Nephrology, Kahramanmaras Sutcu Imam University Faculty of Medicine, Kahramanmaras, Turkey; Division of Nephology, Ankara Bilkent City Hospital, University of Health Sciences, Ankara, Turkey; Division of Nephrology, Istanbul University-Cerrahpasa Cerrahpasa Faculty of Medicine, Istanbul, Turkey; Division of Nephrology, Necmettin Erbakan University Faculty of Medicine, Konya, Turkey; Division of Nephrology, Hamidiye Etfal Training and Research Hospital, University of Health Sciences, Istanbul, Turkey; Division of Nephrology, Ankara Training and Research Hospital, University of Health Sciences, Ankara, Turkey; Division of Nephrology, Marmara University Faculty of Medicine, Istanbul, Turkey; Division of Nephrology, Afyonkarahisar Health Sciences University Faculty of Medicine, Afyonkarahisar, Turkey; Division of Nephrology, Bezmialem Vakif University Faculty of Medicine, Istanbul, Turkey; Division of Nephrology, Bozyaka Training and Research Hospital, University of Health Sciences, Izmir, Turkey; Division of Nephrology, Darica Farabi Training and Research Hospital, University of Health Sciences, Kocaeli, Turkey; Division of Nephrology, Dr Burhan Nalbantoglu State Hospital, Lefkosa, Cyprus; Division of Nephrology, Mersin University Faculty of Medicine, Mersin, Turkey; Division of Nephrology, Tepecik Training and Research Hospital, University of Health Sciences, Izmir, Turkey; Division of Nephrology, Trakya University Faculty of Medicine, Edirne, Turkey; Division of Nephrology, Bursa Uludag University Faculty of Medicine, Bursa, Turkey; Division of Nephrology, Istanbul University Istanbul Faculty of Medicine, Istanbul, Turkey; Division of Nephrology, Gazi University Faculty of Medicine, Ankara, Turkey; Division of Nephrology, Sultan 2. Abdulhamid Han Training and Research Hospital, University of Health Sciences, Istanbul, Turkey; Division of Nephrology, Istanbul University Istanbul Faculty of Medicine, Istanbul, Turkey

**Keywords:** anemia, C3 glomerulopathy, chronicity, CKD, prognosis

## Abstract

**Background:**

Data on the prognostic factors for C3 glomerulopathy (C3G) are limited, and validation of the new C3G histologic index (C3G-HI) in different settings is still needed. We aimed to evaluate the chronicity score of C3G-HI and probable prognostic factors in our population.

**Methods:**

In this registry study, 74 patients from 20 centers with adequate follow-up data were included. Total chronicity score (TCS) was calculated according to percentages of glomerulosclerosis, interstitial fibrosis, tubular atrophy, and presence of arterio- and arteriolosclerosis. Primary composite outcome was defined as doubling of serum creatinine from baseline, undergoing dialysis or transplantation, development of stage 5 chronic kidney disease, or death.

**Results:**

Median age was 34 [interquartile range (IQR) 24–46] years, and 39 patients (52.7%) were male. Median follow-up duration was 36 (IQR 12–60) months, and median TCS was 3 (IQR 1–5). Overall, 19 patients (25.7%) experienced primary composite outcome. Multivariate Cox regression model showed that only hemoglobin [adjusted HR (aHR) 0.67, 95% confidence interval 0.46–0.97, *P *= .035] predicted primary composite outcome, and TCS fell short of the statistical significance (aHR 1.26, 0.97–1.64, *P *= .08). Receiver operating characteristic analysis demonstrated that TCS showed an area under the curve value of 0.68 (0.56–0.78, *P *= .028) in discriminating primary composite outcome at 3 years, and 3-year kidney survival was lower in patients with TCS ≥4 (72.4%) compared with TCS <4 (91.1%) in Kaplan–Meier analysis (*P *= .036).

**Conclusions:**

Low hemoglobin levels predicted dismal outcomes in patients with C3G. TCS ≥4 was associated with a worse 3-year kidney survival, which validated the 3-year prognostic value of the TCS of C3G-HI in our population.

## Graphical Abstract

**Graphical Abstract:**
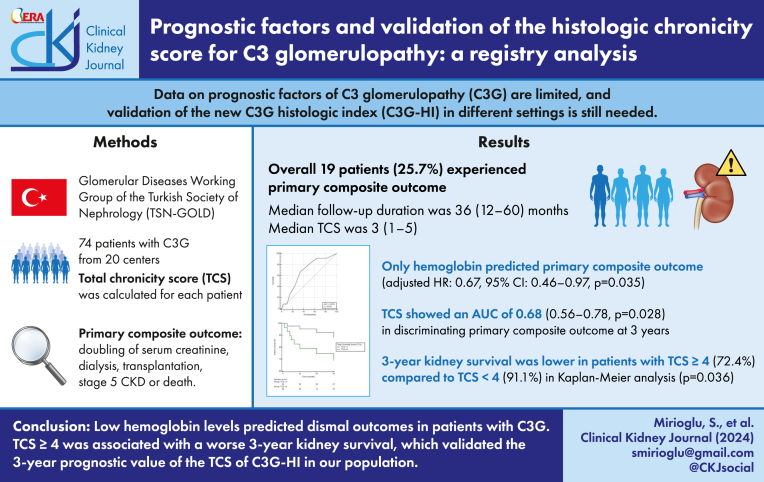

KEY LEARNING POINTS **What was known:**

- Since progression to kidney failure in 5 years has been found in up to half of the patients with C3 glomerulopathy (C3G), it is crucial to select the patients who might really benefit from immunosuppressive therapies.
- A histologic index for C3G (C3G-HI), which includes an activity score consisting of seven parameters and a chronicity score involving four parameters evaluated with a semiquantitative scale, has been developed recently.
- Only one additional cohort examined and validated the usefulness of this tool, further demonstrating that chronicity score had the utmost significance.

**This study adds:**

- Total chronicity score (TCS) fell short of the statistical significance in predicting primary composite outcome, which includes doubling of serum creatinine from baseline, undergoing dialysis or transplantation, development of stage 5 chronic kidney disease or death.
- TCS showed a good area under the curve value in discriminating primary composite outcome at 3 years, and 3-year kidney survival was lower in patients with higher TCS levels.
- Only hemoglobin levels at diagnosis predicted primary composite outcome in multivariate models.

**Potential impact:**

- Three-year prognostic value of the TCS in Turkish population has been validated in this multicenter study.
- TCS of C3G-HI can be used to identify the patients who have high risk of disease progression.

## INTRODUCTION

C3 glomerulopathy (C3G) is a rare kidney disease with an incidence of 1–3 per million, and associated with alternative complement pathway dysregulation in both plasma and glomerular microenvironments [1]. The hallmark histologic finding of C3G is an isolated or dominant C3 deposition, and the disease includes two major subtypes: C3 glomerulonephritis (C3GN) and dense deposit disease (DDD) [1].

Despite the recent advances in delineating the underlying genetic and pathologic mechanisms, treatment options are very few: mycophenolic acid derivatives in combination with glucocorticoids and eculizumab [2, 3]. Their success has been limited in various series, and the data on these treatment options have been mainly observational, lacking randomized controlled trials [3]. Even though the efficacy of mycophenolic acid–based regimens was suggested by two independent cohorts [4, 5], it was not supported by the data from different countries throughout the Europe [6, 7]. Also, eculizumab was not found to be beneficial in patients with a quiescent progressive course instead of a crescentic rapidly progressive disease [8]. As expected, outcomes have been quite dismal in C3G: progression to kidney failure in 5 years has been found in up to half of patients [9]. Thus, it still remains crucial to select the patients who might really benefit from immunosuppressive therapies.

Several predictors of disease progression such as age, estimated glomerular filtration rate (eGFR) and proteinuria at the time of diagnosis, percentage of crescentic and sclerotic glomeruli, and interstitial fibrosis (IF) have been suggested by various studies [6, 7, 10, 11]. Recently, a histologic index for C3G (C3G-HI) has been proposed: an activity score consisting of seven parameters and a chronicity score involving four parameters assessed with a semiquantitative scale [9]. In the original proposal, it was also shown that total activity and chronicity scores could predict the disease progression [9]. To the best of our knowledge, only one additional cohort examined and validated the usefulness of this tool, further demonstrating that chronicity score had the utmost significance [2]. Still, there is an unmet need to evaluate C3G-HI in different settings. Therefore, we aimed to evaluate the chronicity score of C3G-HI and probable prognostic factors in our population.

## MATERIALS AND METHODS

### Patient selection

For the purposes of this nationwide retrospective multicenter study, data were obtained from the registry of the Glomerular Diseases Working Group of the Turkish Society of Nephrology (TSN-GOLD) [12]. Ninety-nine adult patients with biopsy-proven C3G were identified, and 74 patients from 20 centers who were diagnosed between 2000 and 2021 and had follow-up data on treatment strategies, serum creatinine, serum albumin and proteinuria were included. Individuals with C3G associated with systemic autoimmune diseases, monoclonal disorders or viral infections were excluded from the analyses.

Demographic, clinical and laboratory characteristics of all patients were obtained and entered into the registry by an attending nephrologist at every center. Histopathological details were gathered from the individual biopsy data. Hypertension was defined as systolic blood pressure (BP) ≥140 mmHg or diastolic BP ≥90 mmHg or using antihypertensive agents. Urinary protein-to-creatinine ratio in the first morning specimens was used to estimate proteinuria throughout the follow-up, and 24-h urine collection was selectively used to solve any discrepancies arising from the spot urine assessment. Nephrotic-range proteinuria was defined as proteinuria level of ≥3 g/24 h in the absence of nephrotic syndrome. Nephritic syndrome was described as the combination of hypertension, reduced levels of eGFR, microhematuria and subnephrotic proteinuria [2]. eGFRs were calculated by using the Chronic Kidney Disease (CKD) Epidemiology Collaboration (CKD-EPI) 2009 formula [13]. Angiotensin-converting enzyme inhibitors or angiotensin receptor blockers were started in all patients unless they had stage 4 or 5 CKD [14], and these agents were maintained as long as the patients tolerated. Decision to use immunosuppressives and selection of the agents were at the discretion of the treating nephrologist at every center. After 2012, treatment decisions were made based on the Kidney Disease: Improving Global Outcomes guidelines [3, 15].

### Histopathological evaluation and calculation of total chronicity score

A nephropathologist at every center evaluated individual kidney biopsies. In general, adequate kidney biopsy specimens having at least eight glomeruli were assessed by using light and immunofluorescence microscopy. All histochemical and immunofluorescence stains were prepared by using 3–4 µm sections. About 0.4–0.6 cm unfixed tissue was frozen with liquid nitrogen for immunofluorescence staining of immunoglobulin G (IgG), IgM, IgA, C1q, C3, Kappa, Lambda and fibrinogen. Immunofluorescence staining was graded with a semiquantitative scale from 0 to 3 (0, negative; 1, weak; 2, moderate; and 3, strong staining). Remaining tissues were fixed in formalin fixative, embedded in paraffin, and processed routinely for light microscopic evaluation (hematoxylin and eosin, periodic acid–Schiff, methenamine silver–periodic acid, Masson trichrome and Congo red) [16]. C3G diagnosis was made according to the standard criteria, which include a dominant C3 of two or more orders of magnitude more than other immune reactants [17]. Histological data were mainly extracted from the already available biopsy reports. Biopsy reports that belonged to the time before C3G definition were examined by a nephrologist at every center to see whether they met the diagnostic criteria of C3G. If needed, a nephropathologist consultation was made on a case-by-case basis. Finally, the first author (S.M.) examined all histopathological data from the registry and confirmed the diagnoses. IF and tubular atrophy (TA) were graded using a semiquantitative scale from 0 to 3: 0, normal; 1 (mild), <25% of interstitium; 2 (moderate), 25%–50%; and 3 (severe), >50% [16]. Endocapillary proliferation was described as an increase in number of cells within glomerular capillary lumina, causing luminal narrowing or obliteration, and the presence of edema and infiltration of mononuclear cells in nonfibrotic cortex was defined as interstitial inflammation [9]. Total chronicity score (TCS) out of 10 was calculated according to percentages of glomerulosclerosis, IF, TA and presence of arterio- and arteriolosclerosis [9]. In order to calculate the TCS, another semiquantitative scale from 0 to 3 was applied for percentages of glomerulosclerosis, IF and TA, whereas presence of arterio- and arteriolosclerosis was assessed as 0 or 1 [9]. Total activity scores could not be computed since reevaluation of all biopsy samples were not performed.

### Study outcomes

Primary composite outcome was defined as doubling of serum creatinine from baseline, undergoing dialysis or transplantation, development of stage 5 CKD (eGFR <15 mL/min/1.73 m^2^) or death. Secondary outcome was complete or partial remission (CR or PR). CR was determined to be achieved when proteinuria decreased to ≤0.3 g/24 h with normal serum albumin and creatinine concentrations, while PR was considered as a proteinuria reduction of ≥50% (and a proteinuria value of <3 g/24 h in patients with nephrotic syndrome or nephrotic-range proteinuria at baseline) and stabilization or improvement in kidney function [3]. Associations of clinical, laboratory and histopathological features with primary composite outcome were also evaluated.

### Statistical analyses

Results were expressed as mean ± standard deviation when normally distributed or as median [interquartile range (IQR)] otherwise. Comparisons of continuous variables between the groups were evaluated with *t*-tests or the Mann–Whitney *U* test where appropriate. Differences in the proportions of the groups were compared using the chi-squared or Fisher's exact test. Relationships were determined by Pearson correlation coefficient or Spearman's rho. Variables found a promising effect on the primary composite outcome in univariate analyses (a *P*-value of ≤.10 for each variable) were included in the multivariate Cox proportional hazards model. Age, sex and variables which are already known to affect the outcomes were added to the models, as well. Results of the regression models were demonstrated as hazard ratios (HRs) and 95% confidence intervals (CIs). Receiver operating characteristic (ROC) curve was used and area under the curve (AUC) value was calculated to assess the performance of the TCS in discriminating primary composite outcome. Periods of primary outcome-free kidney survival were analyzed using Kaplan–Meier curves by using log-rank test, and this period for each patient was computed from baseline evaluation to the last follow-up, primary composite outcome or a specific timepoint. Statistical analyses were performed with SPSS for Windows (SPSS version 25.0, IBM Corp., Armonk, NY, USA), and graphics were generated using MedCalc for Windows (MedCalc version 19.0, MedCalc Software, Ostend, Belgium). All analyses were two-sided and a *P*-value of ≤.05 was considered as statistically significant.

### Ethical issues

Included patients provided informed consent to extract their data to the registry. The TSN-GOLD registry and the studies derived from its data were approved by Istanbul University Istanbul Faculty of Medicine Ethical Committee (2011/1164), and complied with the Declaration of Helsinki and its later amendments.

## RESULTS

### Baseline features

Median age was 34 (IQR 24–46) years, and 39 patients (52.7%) were male. Thirty-six (48.6%) were hypertensive at the time of diagnosis. Approximately one-third of the whole cohort (26, 35.1%) presented with mixed nephrotic and nephritic features, while 22 (29.7%) had nephrotic syndrome or nephrotic-range proteinuria, 13 (17.6%) nephritic syndrome and 13 (17.6%) had proteinuria (with or without hematuria) with stable eGFR. Median serum creatinine and eGFR levels were 1.3 (IQR 0.7–2.1) mg/dL and 58.9 (IQR 30.9–119.7) mL/min/1.73 m^2^, respectively. Median serum albumin was 3.4 (IQR 2.5–4) g/dL, median proteinuria was 4400 (IQR 1498–6628) mg/day and 71.6% of all patients had hematuria. Mean hemoglobin level was 11.8 ± 2.5 g/dL, and eGFR and hemoglobin were moderately correlated (r = 0.541, *P *< .001). Demographic, clinical and histopathological characteristics of all patients are shown in Table 1.

**Table 1:** Demographic, clinical and histopathological characteristics of all patients at the baseline (*n* = 74).

Characteristics	Values
Sex (male), *n* (%)	39 (52.7)
Age at diagnosis (years), median (IQR)	34 (24–46)
Hypertension at diagnosis, *n* (%)	36 (48.6)
Systolic BP (mmHg), median (IQR)	130 (120–140)
Diastolic BP (mmHg), median (IQR)	80 (74–90)
Presentation, *n* (%)	
Proteinuria with or without hematuria, with stable eGFR	13 (17.6)
Nephrotic syndrome or nephrotic-range proteinuria	22 (29.7)
Nephritic syndrome	13 (17.6)
Mixed (nephrotic and nephritic)	26 (35.1)
Serum creatinine (mg/dL), median (IQR)	1.3 (0.7–2.1)
eGFR (mL/min/1.73 m^2^), median (IQR)	58.9 (30.9–119.7)
Serum albumin (g/dL), median (IQR)	3.4 (2.5–4.0)
Hemoglobin (g/dL), mean ± SD	11.8 ± 2.5
Low C3, *n* (%)	36/71 (50.7)
Low C4, *n* (%)	7/67 (10.4)
Proteinuria (mg/day), median (IQR)	4400 (1498–6628)
Hematuria, *n* (%)	53 (71.6)
Histolopathological features	
Percentage of global and segmental glomerulosclerosis, median (IQR)	10.4 (1.5–30.3)
IF, *n* (%)	
Grade 0	31 (41.9)
Grade 1	28 (37.8)
Grade 2	13 (17.6)
Grade 3	2 (2.7)
TA, *n* (%)	
Grade 0	21 (28.4)
Grade 1	39 (52.7)
Grade 2	10 (13.5)
Grade 3	4 (5.4)
Arterio- and arteriolosclerosis, *n* (%)	28 (37.8)
Endocapillary proliferation, *n* (%)	35 (47.3)
Interstitial inflammation, *n* (%)	53 (71.6)
Presence of crescents, *n* (%)	20 (27)
Cellular and/or fibrocellular crescents, *n* (%)	16 (21.6)

SD: standard deviation.

The median number of glomeruli was 18 (14–31.3). Twenty-one (28.4%) biopsies were performed before the establishment of the diagnostic criteria of C3G in 2013, so these reports were re-examined by a nephrologist at every center and a nephropathologist consultation was made if needed. Median percentage of sclerotic glomeruli was 10.4 (IQR 1.5–30.3). Grade 0, 1, 2 and 3 IF were seen in 31 (41.9%), 28 (37.8%), 13 (17.6%) and 2 (2.7%) patients, respectively. Grade 0, 1, 2 and 3 TA were found in 21 (28.4%), 39 (52.7%), 10 (13.5%) and 4 (5.4%) patients, respectively, as well. Arterio- and arteriosclerosis was noticed in 28 (37.8%) patients, and median TCS was calculated as 3 (IQR 1–5). Twenty (27%) individuals had crescents. In these 20 patients, median percentage of glomeruli with crescents was 10 (IQR 5.6–21.8). Sixteen patients had cellular and/or fibrocellular crescents and 5 had fibrous crescents. No patients had lesions suggesting concomitant thrombotic microangiopathy in the diagnostic biopsy.

### Follow-up and study outcomes

Median follow-up duration of the whole cohort was 36 (IQR 12–60) months. Fifty-five patients (74.3%) were treated with some form of immunosuppression: 55 (74.3%) used glucocorticoids, 25 (33.8%) mycophenolic acid derivatives, 11 (14.9%) cyclophosphamide, 5 (6.8%) eculizumab and 2 (2.7%) rituximab (Table 2).

**Table 2:** Treatment characteristics and study outcomes (*n* = 74).

Treatment	*n* (%)
Any immunosuppression	55 (74.3)
Glucocorticoids	55 (74.3)
Mycophenolic acid derivatives	25 (33.8)
Cyclophosphamide	11 (14.9)
Eculizumab	5 (6.8)
Azathioprine	5 (6.8)
Calcineurin inhibitors	4 (5.4)
Rituximab	2 (2.7)
Outcomes	
Primary composite outcome	19 (25.7)
Dialysis or transplantation	12 (16.2)
Doubling of serum creatinine	4 (5.4)
Stage 5 CKD	1 (1.4)
Death	3^a^ (4.1)
Secondary outcome (remission)	31 (41.9)
CR	14 (18.9)
PR	17 (23)

a One patient died shortly after becoming dialysis-dependent.

Overall, 19 patients (25.7%) experienced primary composite outcome over a median of 24 (IQR 6–51) months. Twelve (16.2%) underwent kidney replacement therapies, four (5.4%) experienced doubling of serum creatinine and one (1.4%) reached stage 5 CKD. Three (4.1%) patients died due to infections (*n* = 2) and unknown causes (*n* = 1), including a patient who died shortly after becoming dialysis dependent. At their last follow-up, 31 patients (41.9%) were in some form of remission (14 CR and 17 PR). Median serum creatinine and eGFR levels reached 1.1 (IQR 0.6–1.8) mg/dL and 67.7 (IQR 40.9–111.9) mL/min/1.73 m^2^, respectively. Also, median serum albumin and proteinuria were 4.1 (IQR 3.4–4.4) g/dL and 1021 (IQR 387.5–3101.5) mg/day, respectively.

Univariate analyses of all patients revealed that presence of cellular and/or fibrocellular crescents (HR 2.95, 95% CI 1.19–7.32, *P *= .020), grade 2–3 TA (HR 2.77, 95% CI 1.03–7.42, *P *= .043), systolic BP (HR 1.02, 95% CI 1.01–1.03, *P *= .003) and diastolic BP (HR 1.07, 95% CI 1.02–1.11, *P *= .002), eGFR (HR 0.99, 95% CI 0.97–0.997, *P *= .011), serum albumin (HR 0.46, 95% CI 0.26–0.81, *P *= .008) and hemoglobin (HR 0.64, 95% CI 0.50–0.82, *P *< .001) at the time of diagnosis predicted the primary composite outcome (Table 3). As TCS and TA have overlapping components, two models were created during multivariate Cox regression. Both models included male sex, age at diagnosis, presence of cellular and/or fibrocellular crescents, systolic and diastolic BP, eGFR, serum albumin, hemoglobin, proteinuria and use of immunosuppression as variables. In addition, grade 2–3 TA was included in Model 1, and TCS in Model 2. The second model demonstrated that only hemoglobin [adjusted HR (aHR) 0.67, 95% CI 0.46–0.97, *P *= .035] predicted the primary composite outcome. Even though they fell short of the statistical significance, a trend was observed for serum albumin (aHR 0.45, 95% CI 0.20–1.004, *P *= .051), systolic BP (aHR 1.03, 95% CI 0.999–1.06, *P *= .06) and TCS (aHR 1.26, 95% CI 0.97–1.64, *P *= .08) (Table 4).

**Table 3:** Univariate regression analysis regarding primary composite outcome in all patients.

		Univariate analysis	
		
Variables	Number (%) of patients with available data for analysis	HR (95% CI)	*P*
Male sex	74 (100)	1.68 (0.66–4.29)	.28
Age at diagnosis	74 (100)	1.02 (0.99–1.06)	.17
Percentage of sclerotic glomeruli	74 (100)	1.004 (0.99–1.02)	.70
Presence of cellular and/or fibrocellular crescents	74 (100)	2.95 (1.19–7.32)	**.020**
Presence of fibrous crescents	74 (100)	0.04 (0.00–53.87)	.39
Endocapillary proliferation	74 (100)	2.06 (0.79–5.37)	.14
Interstitial inflammation	74 (100)	2.41 (0.79–7.36)	.12
Arterio- and arteriolosclerosis	74 (100)	1.50 (0.60–3.74)	.39
Grade 2–3 IF	74 (100)	1.49 (0.48–4.59)	.49
Grade 2–3 TA	74 (100)	2.77 (1.03–7.42)	**.043**
TCS	74 (100)	1.16 (0.97–1.38)	.11
TCS ≥4	74 (100)	1.63 (0.66–4.02)	.29
Systolic BP^a^	74 (100)	1.02 (1.01–1.03)	**.003**
Diastolic BP^a^	74 (100)	1.07 (1.02–1.11)	**.002**
eGFR^a^	74 (100)	0.99 (0.97–0.997)	**.011**
Serum albumin^a^	72 (97.3)	0.46 (0.26–0.81)	**.008**
Hemoglobin^a^	71 (95.9)	0.64 (0.50–0.82)	**<.001**
Proteinuria^a^	74 (100)	1.00 (1.00–1.00)	.32
Proteinuria^a^ (log^b^)	74 (100)	1.13 (0.45–2.87)	.80
Proteinuria ≥3000 mg/day^a^	74 (100)	1.84 (0.66–5.14)	.24
Hematuria^a^	74 (100)	1.47 (0.48–4.47)	.50
Low serum C3^a^	71 (95.9)	1.27 (0.50–3.23)	.61
Low serum C4^a^	67 (90.5)	0.57 (0.08–4.33)	.59
Use of immunosuppression	74 (100)	1.26 (0.36–4.43)	.72
Glucocorticoids	74 (100)	1.26 (0.36–4.43)	.72
Mycophenolic acid derivatives	74 (100)	0.94 (0.36–2.43)	.89
Cyclophosphamide	74 (100)	1.49 (0.49–4.50)	.48
Eculizumab	74 (100)	2.66 (0.59–11.98)	.20
Azathioprine	74 (100)	0.66 (0.09–4.999)	.69
Calcineurin inhibitors	74 (100)	0.04 (0.00–47.38)	.38

a At the time of diagnosis.

b A log_10_ transformation was used.

Statistically significant results were shown in bold values.

**Table 4:** Multivariate Cox regression analyses regarding primary composite outcome in all patients.

	Multivariate analysis Model 1^b^		Multivariate analysis Model 2^b^	
Variables	HR (95% CI)	*P*	HR (95% CI)	*P*
Male sex	1.02 (0.28–3.73)	0.98	1.03 (0.29–3.65)	0.96
Age at diagnosis	0.997 (0.96–1.04)	0.90	1.00 (0.96–1.05)	1.00
Presence of cellular and/or fibrocellular crescents	0.94 (0.27–3.23)	0.92	0.79 (0.23–2.77)	0.72
Grade 2–3 TA	2.76 (0.60–12.72)	0.19		
TCS			1.26 (0.97–1.64)	0.08
Systolic BP^a^	1.03 (0.996–1.06)	0.10	1.03 (0.999–1.06)	0.06
Diastolic BP^a^	0.997 (0.92–1.08)	0.94	0.998 (0.93–1.07)	0.95
eGFR^a^	0.995 (0.98–1.01)	0.52	1.00 (0.99–1.02)	0.93
Serum albumin^a^	0.54 (0.27–1.08)	0.08	0.45 (0.20–1.004)	0.051
Hemoglobin^a^	0.74 (0.53–1.04)	0.09	0.68 (0.46–0.97)	**0.035**
Proteinuria^a^	1.00 (1.00–1.00)	0.40	1.00 (1.00–1.00)	0.46
Use of immunosuppression	1.19 (0.17–8.13)	0.86	0.80 (0.12–5.20)	0.82

a At the time of diagnosis.

b Both models included male sex, age at diagnosis, presence of cellular and/or fibrocellular crescents, systolic BP, diastolic BP, eGFR, serum albumin, hemoglobin, proteinuria and use of immunosuppression as variables. In addition, grade 2–3 TA was included in Model 1, and TCS in Model 2.

Statistically significant results were shown in bold values.

**Figure 1:**
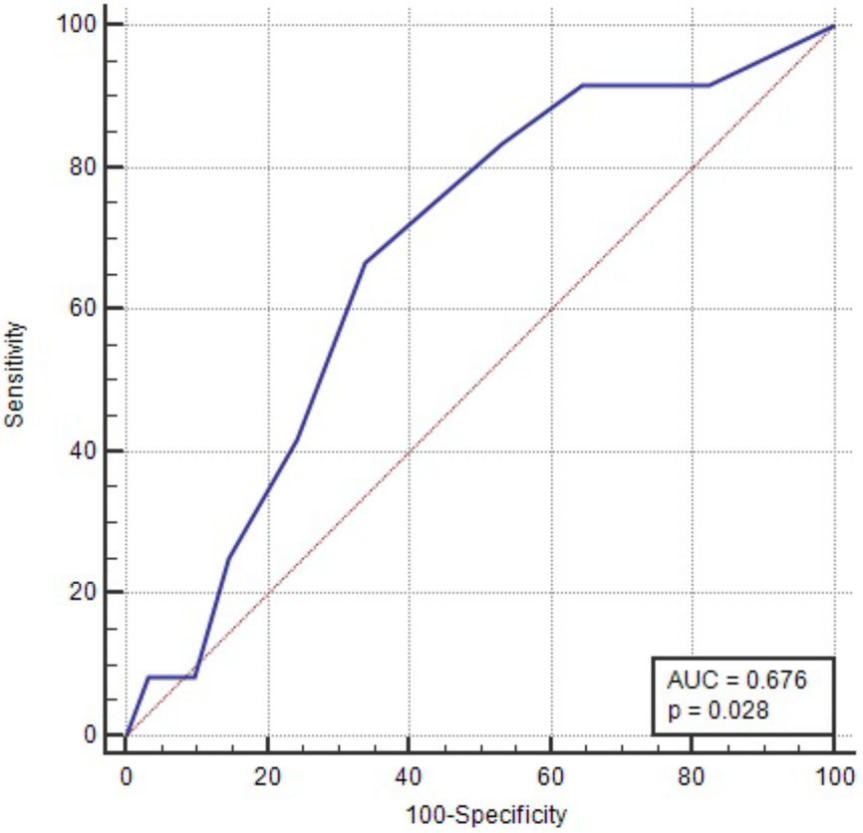
ROC curve showed that total chronicity score demonstrated an AUC value of 0.676 (95% CI 0.557–0.780, *P *= .028) in discriminating primary composite outcome at 3 years. A cut-off of ≥4 had 66.7% sensitivity and 66.1% specificity.

**Figure 2:**
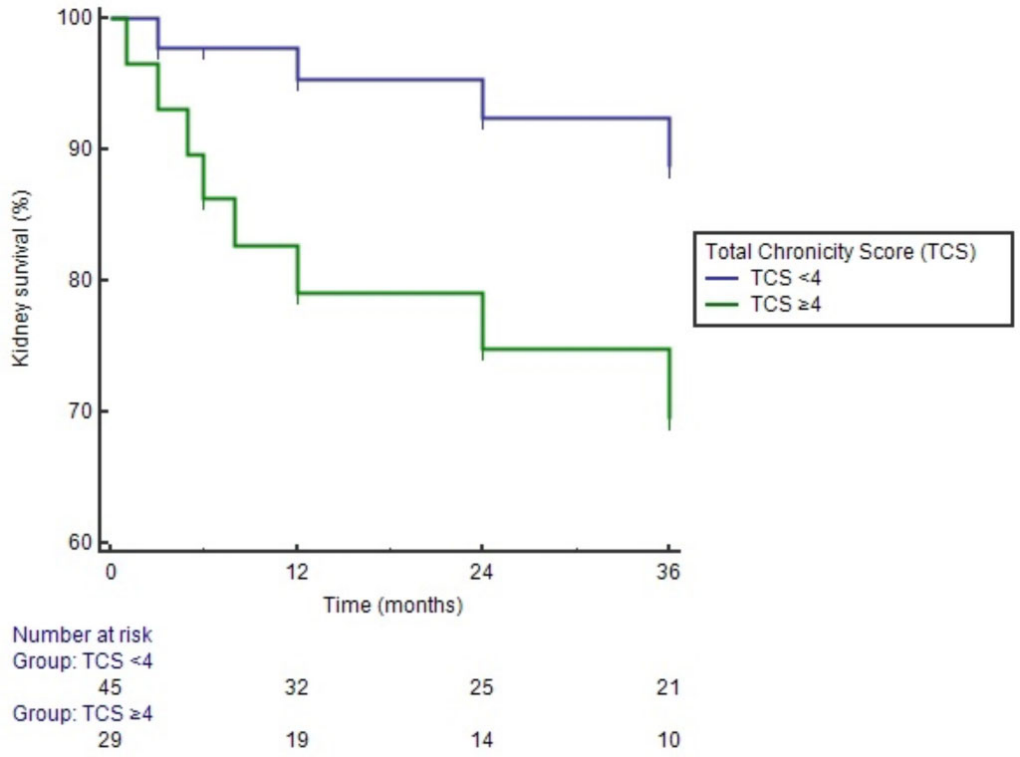
Kaplan–Meier analysis demonstrated that 3-year kidney survival was lower in patients with TCS ≥4 (72.4%) compared with TCS <4 (91.1%) (*P *= .036 with log-rank test).

Since the median follow-up period was 36 months, survival analyses for primary composite outcome at this time were conducted. A ROC analysis demonstrated that TCS showed an AUC value of 0.68 (95% CI 0.56–0.78, *P *= .028) in discriminating primary composite outcome at 3 years (Fig. 1). A cut-off value of ≥4 had 66.7% sensitivity and 66.1% specificity. In addition, Kaplan–Meier analysis revealed that 3-year kidney survival was lower in patients with TCS ≥4 (72.4%) compared with TCS <4 (91.1%) (*P *= .036 with log-rank test) (Fig. 2).

Twenty-nine patients (39.2%) had a TCS of ≥4. When compared with patients with TCS <4, these patients tended to present with mixed nephrotic and nephritic features (*P *= .041). Their median serum creatinine [1.6 (IQR 0.95–2.65) vs 1.1 (IQR 0.65–1.9) mg/dL, *P *= .031] and proteinuria [5000 (IQR 3779–6455) vs 2800 (IQR 1167–6750) mg/day, *P *= .028] levels were higher. Treatment modalities did not differ between these patients, and the study outcomes were generally same. Notably, patients with TCS ≥4 had a higher death rate (10.3% vs 0%, *P *= .028) (Table 5).

**Table 5:** Various features and outcomes of all patients according to the TCS.

Characteristics	TCS <4 (*n* = 45)	TCS ≥4 (*n* = 29)	*P*
Sex (male), *n* (%)	21 (46.7)	18 (62.1)	.24
Age at diagnosis (years), mean ± SD	35 ± 13.2	36.4 ± 14.9	.68
Hypertension at diagnosis, *n* (%)	26 (57.8)	12 (41.4)	.23
Systolic BP (mmHg), median (IQR)	130 (120–140)	130 (115–141)	.68
Diastolic BP (mmHg), median (IQR)	80 (77.5–90)	84 (70–90)	.48
Presentation, *n* (%)			
Proteinuria with or without hematuria, with stable eGFR	11 (24.4)	2 (6.9)	**.041**
Nephrotic syndrome or nephrotic-range proteinuria	13 (28.9)	9 (31)	
Nephritic syndrome	10 (22.2)	3 (10.3)	
Mixed (nephrotic and nephritic)	11 (24.4)	15 (51.7)	
Serum creatinine (mg/dL), median (IQR)	1.1 (0.65–1.9)	1.6 (0.95–2.65)	**.031**
eGFR (mL/min/1.73 m^2^), median (IQR)	83.5 (42.9–121.1)	50.2 (25.6–103.7)	.06
Serum albumin (g/dL), mean ± SD	3.3 ± 0.9	3.2 ± 0.8	.62
Hemoglobin (g/dL), mean ± SD	11.8 ± 2.5	11.9 ± 2.7	.97
Proteinuria (mg/day), median (IQR)	2800 (1167–6750)	5000 (3779–6455)	**.028**
Hematuria, *n* (%)	12 (26.7)	9 (31)	.68
Low C3, *n* (%)	23/42 (54.8)	13/29 (44.8)	.47
Low C4, *n* (%)	5/39 (12.8)	2/28 (7.1)	.45
Histopathological features			
Percentage of global and segmental glomerulosclerosis, median (IQR)	5.6 (0–10.4)	35.3 (18.3–62.4)	**<.001**
IF, *n* (%)			
Grade 0	27 (60)	4 (13.8)	**<.001**
Grade 1	16 (35.6)	12 (41.4)	
Grade 2	2 (4.4)	11 (37.9)	
Grade 3	0 (0)	2 (6.9)	
TA, *n* (%)			
Grade 0	19 (42.2)	2 (6.9)	**<.001**
Grade 1	24 (53.3)	15 (51.7)	
Grade 2	2 (4.4)	8 (27.6)	
Grade 3	0 (0)	4 (13.8)	
Arterio- and arteriolosclerosis, *n* (%)	6 (13.3)	22 (75.9)	**<.001**
Endocapillary proliferation, *n* (%)	20 (44.4)	15 (51.7)	.64
Interstitial inflammation, *n* (%)	29 (64.4)	24 (82.8)	.09
Presence of crescents, *n* (%)	10 (22.2)	10 (34.5)	.29
Cellular and/or fibrocellular crescents, *n* (%)	11 (24.4)	10 (34.5)	.43
Treatment, *n* (%)			
Any immunosuppression	31 (68.9)	24 (82.8)	.28
Glucocorticoids	31 (68.9)	24 (82.8)	.28
Mycophenolic acid derivatives	14 (31.1)	11 (37.9)	.62
Cyclophosphamide	5 (11.1)	6 (20.7)	.26
Eculizumab	4 (8.9)	1 (3.4)	.36
Azathioprine	4 (8.9)	1 (3.4)	.36
Calcineurin inhibitors	2 (4.4)	2 (6.9)	.65
Rituximab	1 (2.2)	1 (3.4)	.75
Outcomes, *n* (%)			
Primary composite outcome	10 (22.2)	9 (31)	.40
Dialysis or transplantation	7 (15.6)	5 (17.2)	.85
Doubling of serum creatinine	3 (6.7)	1 (3.4)	.55
Stage 5 CKD	0 (0)	1 (3.4)	.21
Death	0 (0)	3^a^ (10.3)	**.028**
Secondary outcome (remission)	21 (46.7)	10 (34.5)	.34
CR	11 (24.4)	3 (10.3)	.31
PR	10 (22.2)	7 (24.1)	
No remission	24 (53.3)	19 (65.5)	

SD: standard deviation.

a One patient died shortly after becoming dialysis-dependent.

Statistically significant results were shown in bold values.

## DISCUSSION

In recent years, we have witnessed great developments in the area of glomerular diseases, especially in enlightening the pathobiology of various diseases, which culminated in a better understanding, and hence a better classification of these ailments. Nevertheless, therapeutic options for most diseases are still scarce. It is of utmost importance to delineate the prognostic factors of the patients in order to not only envision the disease course but also choose the patients who will likely benefit from immunosuppressive agents. As a result of this search for C3G, Bomback *et al*. developed the C3G-HI for prognostic purposes in 2018 [9]. In this study, we found that TCS of this histologic index ≥4 was able to discriminate a worse prognosis at 3 years. Also, we demonstrated that hemoglobin level at the time of diagnosis could predict the outcomes, and none of the immunosuppressive agents was associated with better results.

Over the last decade, cohorts from various countries have identified different prognostic factors for C3G: age, eGFR, proteinuria level, presentation with nephrotic syndrome, disease subtype (DDD), crescents, percentage of sclerotic glomeruli, degree of IF and TA, presence of certain genetic variations and autoantibodies for complement proteins, and treatment with immunosuppressives [2, 6, 7, 9–11]. Notably, traditional histopathologic features reflecting chronicity were generally among the predictors in most of these studies [2, 6, 9, 11]. TCS appeared to be a better predictor than the total activity score in the original study [9], and a validation cohort showed that only TCS was able to discriminate the patients with worse outcomes [2]. Similar findings have been reported in other glomerular diseases like lupus nephritis and IgA nephropathy (IgAN) [18–20]. For instance, T2 score, which is the presence of IF and/or TA in over 50% of the cortical area, has been noted as the strongest predictor of the Oxford Classification for IgAN [19]. In this study, we validated prognostic use of the TCS at 3 years in our population. Although it was successful at the 3-year timepoint, it could not differentiate the primary composite outcome throughout the whole follow-up as the analysis may have been hindered by lack of long-term data of many patients. There was a certain trend in the regression model, so limited number of patients compared with previous studies might have played a role in the lack of statistical significance, as well [2, 9]. Also, we did not report the results of the total activity score since this could only have been done by reevaluation of all biopsy samples, which could not be performed within the scope of a registry study.

Oxygen delivery to the kidneys is hindered in the presence of anemia, and this hypoxia may increase fibrosis of the kidneys aggravating the hypoxia itself, which may result in a dangerous cycle leading to kidney failure [21]. Although anemia is a known complication of CKD, clinical evidence suggests that it can also be a risk factor for disease progression [22]. Notably, various studies in IgAN reported worse outcomes in patients with anemia [23–25]. We showed that low hemoglobin levels were associated with dismal outcomes in patients with C3G, which is in line with these findings.

Serum albumin and systolic BP seemed to have some value in predicting the primary composite outcome in our study. Serum albumin has the potential to reflect the time-averaged proteinuria, which is known to account for the prognostic effects of proteinuria over time in studies of glomerulonephritis [26, 27]. Also, serum albumin might reveal the inflammatory status of a patient [27]. These two features might explain why serum albumin was found to have some form of prognostic value in our study while proteinuria at diagnosis was not. Since hypertension is a known risk factor of CKD progression [28], our findings with regard to systolic BP were very much expected. Unfortunately, we did not have longitudinal data on BP, thus we were not able to analyze the effects of the longitudinal BP trajectory on the outcomes of patients with C3G [29].

A quarter of our patients reached the primary composite outcome, and 40% were in some form of remission at the end of follow-up. Our remission rates were generally in line with the previous reports [9], but kidney failure was reported to be as high as 40% in contemporary papers of C3G [2, 9]. This difference could be partly related to the limited median duration of follow-up in our study. Still, another cohort from our population demonstrated similar results, so this difference may have partially originated from genetic and environmental backgrounds [6]. Also, various immunosuppressive treatment approaches did not seem to show efficacy in our study. Glucocorticoids and mycophenolic acid derivatives were used in 74% and 33% of the patients, respectively; however, eculizumab was given to only five patients. Immunosuppressive use was numerically higher in patients with TCS ≥4 (82.8% vs 68.9%), which may have played a role in the lack of response. Moreover, Caliskan *et al*. analyzed 66 patients with C3G according to treatment strategies, and found no differences in outcomes between two immunosuppressive regimens and conservative care [6]. Inherent features of our population might have interfered with the response rates, as well.

Our study suffered from several limitations. First, this is a retrospective observational study, thus a cause–effect relationship cannot be established. Second, we could not perform a centralized pathology review for biopsy samples. TCS was calculated by using already available biopsy data, and total activity scores could not be computed. Third, we did not provide information on antibodies for complement proteins or genetic analysis, since most of our patients lacked results of these tests, which are not easy to perform in resource-limited settings. Fourth, dichotomization of C3G into C3GN and DDD subtypes was not reported to the registry in most of the patients, and therefore was not included in the analyses. Fifth, duration of follow-up was limited. Sixth, we did not have longitudinal data on BP. Finally, even though we performed adjusted analyses, prognostic role of possible unmeasured confounders cannot be overlooked. On the other hand, our study has some strengths. To the best of our knowledge, this is the second report on external validation of C3G-HI. Also, we provided the largest multicenter cohort of C3G from our country.

In conclusion, we demonstrated that low hemoglobin levels predicted dismal outcomes in patients with C3G. TCS ≥4 was associated with a worse 3-year kidney survival, which validated the prognostic value of the TCS of C3G-HI at 3 years in our population. Further studies are awaited to evaluate the C3G-HI in different populations.

## Data Availability

Deidentified data are available upon reasonable request from the corresponding authors.
